# Impact of Chromosomal Instability and Aneuploidy in Cancer Development

**DOI:** 10.1146/annurev-cancerbio-071124-101613

**Published:** 2025-09-30

**Authors:** Amanda K. Mennie, Brittiny Dhital, Peter Ly

**Affiliations:** 1 Children’s Medical Center Research Institute, University of Texas Southwestern Medical Center, Dallas, Texas, USA; 2 Department of Pathology, University of Texas Southwestern Medical Center, Dallas, Texas, USA; 3 Department of Internal Medicine, Division of Hematology and Oncology, University of Texas Southwestern Medical Center, Dallas, Texas, USA; 4 Department of Cell Biology, Department of Pediatrics, Harold C. Simmons Comprehensive Cancer Center, University of Texas Southwestern Medical Center, Dallas, Texas, USA

**Keywords:** aneuploidy, chromosomal instability, cell division, chromosome segregation, cell cycle, cancer genome

## Abstract

Somatic human cells contain a diploid genome consisting of 23 pairs of chromosomes. The maintenance of this diploid state is essential across all layers of biological organization, ranging from the physiology of individual cells to the proper regulation of tissue homeostasis and organismal development. Most cancer cells, however, harbor an aneuploid genome with an abnormal number of chromosomes, including whole and/or partial chromosome gains and losses. These alterations arise as a consequence of mitotic chromosome segregation errors and/or ongoing chromosomal instability (CIN). While aneuploidy usually imposes a fitness cost to nontransformed cells, certain recurrent aneuploidies confer adaptive advantages that are subjected to positive selection throughout tumorigenesis. In this review, we discuss how aneuploidy impacts cellular physiology, fitness, and adaptability in the context of cancer development. We also examine how the aneuploid state and CIN can create vulnerabilities that may be exploited for therapeutic intervention.

## INTRODUCTION

Aneuploidy is defined as an abnormal number of chromosomes that deviate from the diploid (2N) complement. This encompasses the gain and loss of entire chromosomes, as well as structural abnormalities involving chromosome arms and large chromosomal segments. These megabase-sized DNA copy number alterations are a pervasive feature of human cancer genomes (Sdeor et al. 2024). In somatic cells, aneuploidy is caused by defects during mitotic cell division in which one or more chromosomes are unequally inherited between daughter cells. The term chromosomal instability (CIN) refers to an elevated rate of such chromosome segregation errors, leading to ongoing karyotypic diversity within a cell population (Lengauer et al. 1997). While CIN is a major driver of aneuploidy, it is important to distinguish between these two concepts: Not all aneuploid cells exhibit CIN. For example, many tumors maintain a stable yet abnormal karyotype without evidence of continuing mitotic errors, highlighting a key distinction between aneuploidy as a state and CIN as a dynamic process (van Jaarsveld & Kops 2016).

Beyond cancer, aneuploidy can also arise early during human development, where it is poorly tolerated and represents the leading cause of reproductive failure, spontaneous miscarriage, and congenital disorders (Milan 2025, Nagaoka et al. 2012). Only a limited subset of aneuploidies—most notably trisomy of chromosomes 13, 18, 21, or the sex chromosomes—are compatible with live birth, and these are often accompanied by severe developmental abnormalities. Certain trisomies, such as trisomy 8, may be observed at high levels of mosaicism. Although these alterations are not uniformly present in all cells of live-born individuals, the persistence of a substantial fraction of trisomic cells implies incomplete selective elimination during development. This review examines our current understanding of how aneuploidy and CIN contribute to malignant transformation, with a focus on the cellular consequences and evolutionary pressures that enable cancer cells to tolerate, adapt to, and ultimately exploit these genomic alterations throughout tumorigenesis.

## ANEUPLOIDY AND CHROMOSOMAL INSTABILITY IN CANCER

Aneuploidy is detected in over ~80% of all human cancers with high prevalence in solid tumors (~90%) and hematologic malignancies (~75%) (Weaver & Cleveland 2006). Recent pan-cancer analyses of >10,000 tumors revealed a wide range of aneuploidy frequencies across diverse cancer types, including nearly all glioblastomas, uterine carcinosarcomas, and testicular germ cell tumors harboring aneuploid karyotypes (Taylor et al. 2018). Notably, chromosome arm–level aneuploidies are frequent with recurrent gains of chromosome arms 1q, 7p, 7q, 8q, and 20q and loss of 17p being among the most pervasive events across tumor types (Shih et al. 2023). Several important oncogenic drivers are located on recurrently gained chromosomes, including *EGFR* and *MET* on chromosome 7 and *MYC* on chromosome 8q. The deletion of chromosome 17p, which harbors the tumor suppressor gene *TP53*, results in loss of heterozygosity and subsequent deregulation of critical genome maintenance pathways. These nonrandom patterns of chromosomal gains and losses reflect selective pressures that favor specific karyotypes during tumor evolution (Shih et al. 2023).

Although some aneuploidies are broadly shared across cancers, others show strong tissue specificity. For example, gain of chromosome 21 is common in hematologic malignancies but is often lost in solid tumors, consistent with epidemiological evidence that individuals with Down syndrome are predisposed to leukemia yet relatively protected from solid tumors (Izraeli et al. 2007, Laurent et al. 2020, Marderstein et al. 2024). Similarly, chromosome 13q is recurrently deleted in many cancers but paradoxically gained in colorectal carcinomas (Cancer Genome Atlas Network 2012). Tumors arising from epithelial tissues often acquire an additional copy of chromosome 1q (e.g., lung, breast, liver), whereas squamous cell carcinomas from various tissues are enriched for chromosome 3p loss (Taylor et al. 2018). These distinct patterns suggest that the diverse biological consequences of aneuploidy can be shaped by tissue context and potentially by developmental lineage. In some cases, recurrent chromosome-level alterations appear to function in a manner analogous to mutations in classical oncogenes or tumor suppressors. For instance, gain of chromosome 1q—which results in overexpression of MDM4 and suppression of p53 activity—is mutually exclusive with *TP53* loss in multiple cancer types (Girish et al. 2023), indicating that specific aneuploidies can drive tumorigenesis through dosage effects that substitute for or complement genetic mutations.

Accumulating evidence suggests that aneuploidy is not restricted to advanced stages of malignancy but can emerge early in tumorigenesis. Aneuploid cells have been found in precancerous lesions and even in histologically normal tissues from aged individuals (Lin et al. 2024). In the breast, for example, gain of chromosome 1q and loss of 16q can be detected in morphologically normal ducts and lobules years before the onset of overt malignancy, suggesting that specific aneuploidies may act as initiating events that precondition tissues for transformation (Nishimura et al. 2023). Similar early aneuploid events, such as gain of chromosome 7 and loss of 9p or 10, have been identified in glioblastoma years before clinical diagnosis (Körber et al. 2019). In clear cell renal cell carcinoma (ccRCC), the near-universal loss of chromosome 3p precedes tumor development by decades, often arising during adolescence and prior to somatic mutation of common tumor suppressor genes (Gerlinger et al. 2012, 2014; Mitchell et al. 2018; Zbar et al. 1987). These findings support a model in which aneuploidy serves as an early driver of clonal expansion that lays the foundation for the acquisition of additional genetic events during cancer development.

On the other hand, aneuploidy is also implicated in later stages of tumor progression and contributes to metastasis, disease recurrence, therapeutic resistance, and/or poor prognosis (Davoli et al. 2017, Spurr et al. 2022, Zhao et al. 2025). This is exemplified by therapy-related acute myeloid leukemia, which arises after exposure to chemotherapy and frequently harbors de novo loss of chromosomes 5 and 7 or gain of chromosome 8—karyotypic features that are linked to poor survival (Kayser et al. 2011). In multiple myeloma and hepatocellular carcinoma, gain of chromosome 1q and loss of 8p, respectively, are associated with more aggressive disease and limited treatment options (Roessler et al. 2012, Shah et al. 2017). In prostate cancer, aneuploidy is detectable in preneoplastic lesions and increases with progression to metastatic disease, where specific alterations (e.g., gain of 7, 8q, or X) predict resistance to androgen deprivation therapy (Holcomb et al. 2009, Visakorpi et al. 1995).

The prognostic significance of aneuploidy is further supported by studies linking CIN to clinical outcomes across multiple cancer types. Elevated CIN is implicated in progression to metastatic disease (Bakhoum et al. 2018, Li et al. 2023) and generally associated with poor prognosis (Carter et al. 2006, Kronenwett et al. 2004, Nakamura et al. 2003). However, this relationship is not strictly linear. For instance, high CIN levels are associated with favorable outcomes in triple-negative breast cancer yet predict worse prognosis in hormone receptor–positive subtypes (Birkbak et al. 2011, Roylance et al. 2011). Additionally, in some contexts, both low and high levels of CIN, or the presence of specific aneuploidies, have been correlated with improved patient outcomes (Andor et al. 2016, Birkbak et al. 2011, Jamal-Hanjani et al. 2015, Sansregret et al. 2017, Silk et al. 2013, Vasudevan et al. 2020). These findings suggest that moderate levels of CIN may promote clonal evolution and adaptation, whereas excessive genomic instability may impair cellular fitness and compromise tumor viability. In support of this, genetically engineered mouse models with tunable levels of CIN show that moderate CIN promotes tumor progression in some tissue contexts, whereas low or high levels of CIN do not (Hoevenaar et al. 2020). Insights from single-cell sequencing studies further revealed that aneuploidy can arise in punctuated bursts during tumor evolution (Wang et al. 2014). Notably, resistant and metastatic clones frequently emerge from preexisting aneuploid subpopulations within the primary tumor, underscoring the importance of karyotypic diversity as a reservoir for therapy resistance and disease progression (Yates et al. 2015).

## IMPACT OF ANEUPLOIDY AND CHROMOSOMAL INSTABILITY IN NORMAL CELLS

What causes aneuploidy? During mitotic cell division, the duplicated genome condenses into discrete chromosomes (pairs of sister chromatids) that attach to a bipolar mitotic spindle. Individual chromosomes then align at the metaphase plate before undergoing segregation into two genetically identical daughter cells. The diploid state is largely maintained by the spindle assembly checkpoint (SAC), which ensures equal distribution of genetic material by monitoring kinetochore–microtubule attachments (Lampson & Grishchuk 2017, Lara-Gonzalez et al. 2021, Musacchio & Salmon 2007). Unattached kinetochores trigger the formation of the mitotic checkpoint complex, which signals to inhibit anaphase onset (McAinsh & Kops 2023, Musacchio 2015). Once all chromosomes are correctly attached during metaphase, the anaphase promoting complex/cyclosome (APC/C), an E3 ubiquitin ligase, is activated to degrade securin and cyclin B1. This releases the protease separase, allowing cleavage of cohesin rings that hold together sister chromatids and enabling their segregation to opposite spindle poles (Michaelis et al. 1997, Waizenegger et al. 2002). The nuclear envelope subsequently reforms around the newly separated genomic masses to generate a single diploid nucleus per daughter cell. In this section, we discuss the immediate and long-term consequences that occur following defects in the process of mitotic chromosome segregation.

### Immediate Consequences of Mitotic Errors

Multiple sources of defects can arise during mitosis to compromise the fidelity of chromosome segregation, including sister chromatid cohesion defects, centrosome amplification, SAC inactivation, improper kinetochore–microtubule attachments, premature mitotic exit, and cytokinesis failure (Barber et al. 2008, Cahill et al. 1998, Ganem et al. 2009, Hartwell & Weinert 1989, Hodges et al. 2005, Lens & Medema 2019, Manning et al. 2010, Nigg 2002, Silkworth et al. 2009, Subramanian & Bickel 2008, Thompson & Compton 2008). These defects can cause one or more chromosomes to undergo improper sister chromatid disjunction, improperly align during metaphase, and/or lag behind during anaphase. Such mis-segregated chromosomes may undergo one of several potential fates depending on their spatial position at the completion of mitosis. First, chromosomes can mis-segregate into the nucleus of the incorrect daughter cell, giving rise to aneuploid progeny. Second, lagging chromosomes that are entrapped in the cleavage furrow become damaged during cytokinesis, which can develop into arm-level structural aneuploidies (Janssen et al. 2011). Third, mis-segregated chromosomes can become encapsulated within abnormal nuclear structures called micronuclei, where they often acquire additional DNA damage and replication defects in either the correct or incorrect daughter cell (Mazzagatti et al. 2024). Altogether, these defects can result in aneuploid daughter cells that trigger a cascade of downstream signaling events that culminate in apoptosis, cell cycle arrest, or continued proliferation in a genomically unstable state.

### Activation of the p53 Checkpoint Pathway

In response to errors in mitosis, one or both daughter cells typically activate the p53 pathway (Andreassen et al. 2001, Lanni & Jacks 1998, Marques & Kops 2023, Minn et al. 1996, Thompson & Compton 2010). Although the exact cause of p53 activation remains incompletely understood, recent studies have implicated prolonged mitotic timing, DNA damage, nuclear envelope defects, and the aneuploid state itself as potential sources of checkpoint activation. Human cells normally spend ~60 min in mitosis irrespective of the total length of the cell cycle (Araujo et al. 2016). Extended mitotic duration is indicative of potential problems in mitosis, and—beyond a certain threshold—it can induce p53-mediated apoptotic cell death in mitosis or a partial apoptotic response that triggers caspase-dependent DNA damage followed by cell cycle arrest in the subsequent G1 phase (Orth et al. 2012).

Cells that bypass these apoptotic routes can nevertheless undergo G1 arrest, supporting the activation of cell cycle checkpoints that operate independently of detectable DNA damage. Indeed, this checkpoint in response to prolonged mitotic arrest is governed by the interplay of MDM2 (Fulcher et al. 2025), a ubiquitin ligase that targets p53 for degradation, and USP28, a deubiquitinase that stabilizes it. MDM2 levels decline during mitosis due to its short half-life as USP28 conversely accumulates. Once a critical threshold is reached during a prolonged mitosis, PLK1 facilitates the formation of the 53BP1-USP28-p53 mitotic stopwatch complex. This complex is retained in the daughter cells and elicits a p53-mediated G1 arrest independent of DNA damage, thereby preventing the proliferation of cells that had potentially experienced a problematic mitosis (Fong et al. 2016; Lambrus et al. 2016; Meitinger et al. 2016, 2024). This memory checkpoint functions as a tumor-suppressive barrier that prevents the potential propagation of aneuploid cells. Alternatively, during prolonged mitotic arrest, cells may also undergo mitotic slippage—that is, escape from mitosis without genome segregation due to the gradual degradation of cyclin B over time (Brito & Rieder 2006). Mitotic slippage generates a single tetraploid daughter cell containing a doubling of the genomic content (see the section titled Adaptation via Whole-Genome Duplication).

Direct and indirect sensing of chromosome segregation errors may also activate a p53 response. For example, histone H3.3 on mis-segregated chromosomes can undergo phosphorylation on serine 31, which can rapidly propagate across chromatin in the resulting daughter cells and trigger p53 activation (Hinchcliffe et al. 2016). Blocking histone H3.3 serine 31 phosphorylation using antibodies was sufficient to prevent p53 accumulation and enable aneuploid daughter cells to continue cycling (Hinchcliffe et al. 2016). Additionally, mitotic errors can generate daughter cells with abnormally shaped nuclei, which in turn triggers p53 activation and the accumulation of p21 within hours after telophase, leading to cell cycle arrest in the next interphase through an mTORC2- and ATR-dependent pathway (Hervé et al. 2025). This mechanosensing checkpoint can be activated by mis-segregated chromosomes even in the absence of detectable DNA damage or extended mitotic duration, and instead it correlates with aberrant nuclear morphology driven by altered interactions between heterochromatin and the nuclear lamina (Hervé et al. 2025).

Given the central role of p53 activation in response to mitotic errors (among other cellular stresses), it is therefore unsurprising that *TP53* inactivation is the most frequent and often earliest genetic alteration in cancer (Muller & Vousden 2013). This correlation may reflect either increased rates of chromosome mis-segregation or enhanced survival of aneuploid cells, although the precise contribution of each remains unclear. Even in p53-proficient cells, the mis-segregation of a single chromosome can be tolerated, whereas more extensive aneuploidy or damaged chromosomes resulting from mitotic defects often trigger p53-dependent cell cycle arrest (Santaguida et al. 2017, Soto et al. 2017). To proliferate in the face of persistent CIN, however, cancer cells must overcome this critical checkpoint barrier that normally restrains cell cycle progression in response to genomic perturbations. Disruption of the p53 pathway thus represents a central mechanism to eliminate a critical sensor of DNA damage and an essential effector of cell cycle arrest, thereby permitting cells with extensive genomic abnormalities to continue proliferating (Hafner et al. 2019).

### Proliferative Disadvantage of Aneuploidy

Aneuploidy imposes a proliferative disadvantage in perhaps all model organisms studied to date, including yeast, flies, mice, and mammalian cells, particularly under optimal growth conditions (Lindsley et al. 1972, Niwa et al. 2006, Taylor et al. 2018, Torres et al. 2007, Williams et al. 2008). Yeast cells harboring an extra chromosome proliferate slower than their euploid counterparts, which correlates with gene dosage imbalances driven by the aneuploid chromosome rather than the loss or gain of specific growth-related genes (Donnelly & Storchová 2014). Introducing transcriptionally silent extra chromosomes into yeast does not impair proliferation, implicating transcriptional and/or proteomic burden rather than the presence of additional DNA as a key contributor (Torres et al. 2007). Similar proliferative defects have also been observed in trisomic mouse embryonic fibroblasts and human Down syndrome fibroblasts, which can be reversed by experimentally correcting the karyotype (Li et al. 2012, Segal & McCoy 1974, Williams et al. 2008).

### Gene Dosage and Proteomic Imbalances

The gene dosage hypothesis posits that DNA copy number alterations lead to proportional changes in RNA and protein levels, disrupting the normal stoichiometry of protein complexes and thereby impairing cellular homeostasis (Torres et al. 2007, Williams et al. 2008). Transcriptomic and proteomic analyses have confirmed that, for most genes, elevated chromosome copy number indeed correlates with gene expression changes in aneuploid cells (Dephoure et al. 2014, Kahlem et al. 2004, Pavelka et al. 2010, Stingele et al. 2012, Torres et al. 2010, Upender et al. 2004). However, increased levels of protein subunits belonging to multiprotein complexes are often degraded in an attempt to maintain complex stoichiometry at the cost of energy and protein homeostasis (Liu et al. 2017). This imbalance can induce proteotoxic stress. While heat shock proteins and the unfolded protein response are canonical mechanisms for managing proteomic imbalances (Balch et al. 2008, Hipp et al. 2019, Morimoto 2008), aneuploid cells can also activate autophagy pathways, including p62-mediated sequestration and degradation of misfolded proteins (Stingele et al. 2012). Aneuploid yeast and human cells are therefore hypersensitive to inhibition of autophagy- and proteasome-related pathways (Ariyoshi et al. 2016, Brennan et al. 2019, Donnelly et al. 2014, Ohashi et al. 2015, Oromendia et al. 2012, Santaguida et al. 2015, Stingele et al. 2012, Tang et al. 2011, Torres et al. 2010), suggesting the existence of general aneuploidy vulnerabilities regardless of the identity of the affected chromosome.

**Figure 1:**
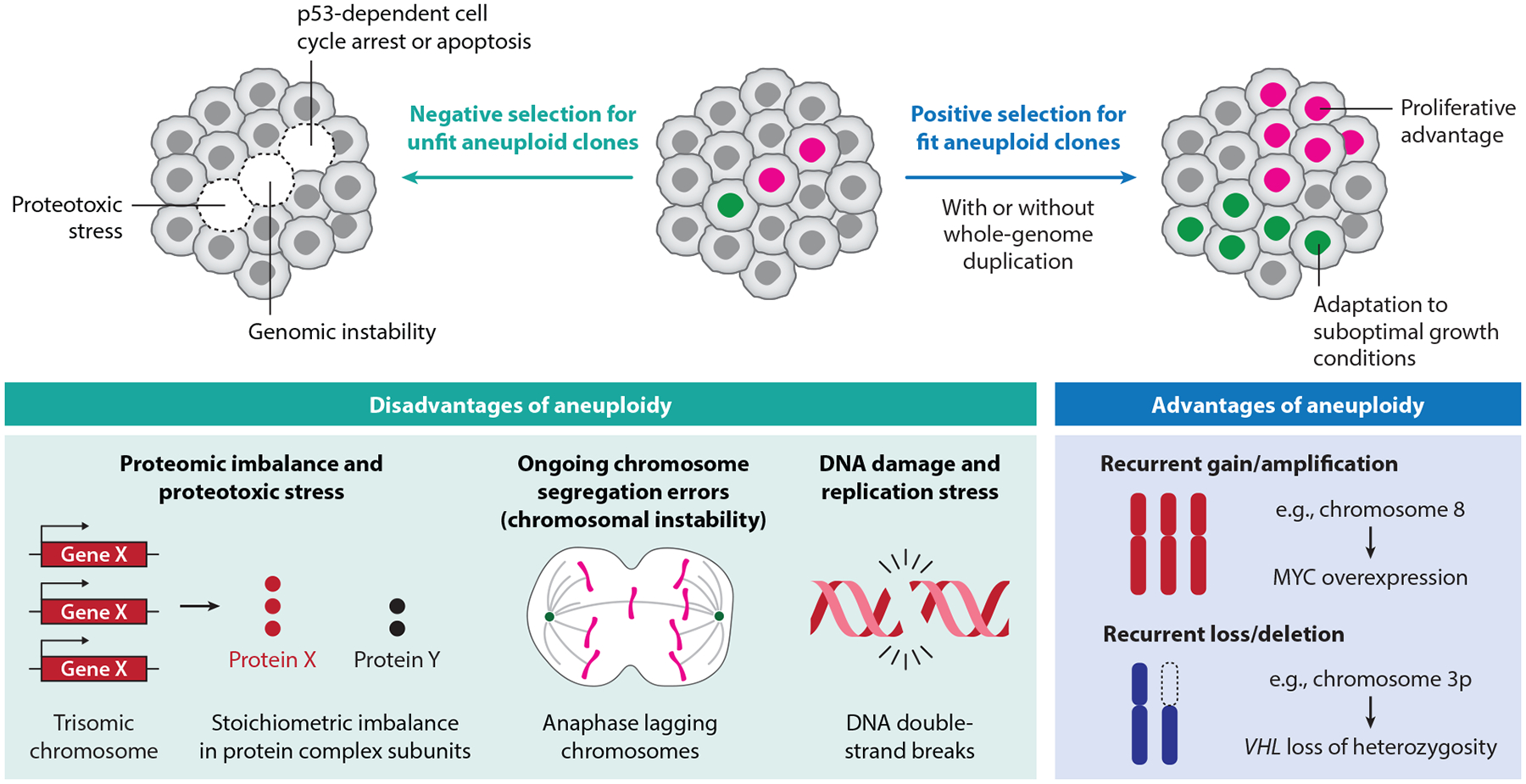
Aneuploidy imposes selective pressures that shape the cancer genome landscape. Aneuploid cells (*colored nuclei*) are subjected to opposing selection forces. Most are outcompeted by diploid cells (*gray nuclei*) due to the fitness costs associated with aneuploidy, including altered gene dosage, proteotoxic stress, cell cycle arrest, apoptosis, and ongoing genomic instability. However, in certain contexts, aneuploid cells can undergo positive selection owing to traits that confer a survival or proliferative advantage, such as enhanced adaptation to different growth conditions, oncogene amplification, or tumor suppressor loss. These adaptive clones may expand and contribute to tumor progression, especially after a whole-genome duplication event that buffers against the detrimental effects of aneuploidy.

Aneuploidy simultaneously alters the abundance and stoichiometry of thousands of genes, leading to imbalances in gene expression and protein complex assembly that can be deleterious to cell fitness (Zhu et al. 2018) (Figure 1). To mitigate these effects, cancer cells can engage dosage compensation mechanisms that attenuate the transcriptional and translational consequences of aneuploidy. Analyses of >300 human cancer cell lines revealed that the expression levels for some genes deviate from expectations based on chromosome copy number, particularly for genes involved in protein complexes (Schukken & Sheltzer 2022). These findings suggest that dosage compensation is selectively and functionally constrained, allowing cancer cells to buffer imbalances in critical pathways while tolerating broader genomic disruptions. A key source of aneuploidy-induced stress is proteotoxicity arising from unbalanced production of complex subunits and increased protein aggregation. To cope with this burden, aneuploid cells activate pathways involved in RNA turnover, translational repression, and protein degradation (Donnelly et al. 2014, Heller et al. 2025, Ippolito et al. 2024, Tang et al. 2011, Torres et al. 2010). These compensatory programs help maintain protein homeostasis in the face of extensive chromosomal abnormalities, supporting the continued survival and proliferation of aneuploid cancer cells.

### Aneuploidy and Ongoing Genomic Instability

Can aneuploidy itself drive ongoing genomic instability and CIN? Studies in budding yeast, fission yeast, and human cells have shown that the presence of an extra chromosome or CIN increases spontaneous DNA damage, replication stress, and/or chromosome segregation defects (Burrell et al. 2013, Garribba et al. 2023, Passerini et al. 2016, Sheltzer et al. 2011) (Figure 1). Cells with extra chromosomes may face increased demands for DNA replication and require elevated nucleotide pools to support their higher chromosomal load. Aneuploid human mammary epithelial cells are particularly dependent on pyrimidine biosynthesis, and when restricted to nucleotide salvage pathways alone, they undergo p53 activation, arrest in S-phase, and heightened sensitivity to DNA damaging agents (Magesh et al. 2025). The introduction of additional chromosomes into diploid human cells by microcell-mediated chromosome transfer further supports the idea that an aneuploid state can promote CIN by elevating the frequency of anaphase lagging chromosomes (Nicholson et al. 2015). Consistent with these findings, highly aneuploid Chinese hamster embryo cells display increased rates of chromosome segregation errors compared to near-diploid counterparts (Duesberg et al. 1998). One possible explanation for these observations is that aneuploid cells are better equipped to tolerate further karyotypic changes, which allows CIN to persist. However, it is worth reemphasizing that many cancers can maintain a stable aneuploid karyotype without evidence of ongoing CIN.

## THE ANEUPLOIDY PARADOX

If aneuploidy impairs cell proliferation, it would be expected to suppress—rather than promote—tumor development. However, the pervasiveness of aneuploidy in human cancer raises a contradiction that was first recognized over a century ago (Boveri 1914, Hansemann 1890) and still remains under debate (Weaver & Cleveland 2006). This is the so-called aneuploidy paradox: Despite its deleterious effects, aneuploidy appears to be a general feature of most cancer genomes.

### Selective Advantage of Aneuploidy

Recurrent aneuploidies are frequently observed in the earliest stages of tumorigenesis, suggesting that in certain contexts, they can confer some selective advantage to cancer cells (Paterson et al. 2020). This context dependency may be central to resolving the aneuploidy paradox. In model systems of yeast, mice, and human cells, specific chromosome gains and losses arise as an adaptive response to intrinsic and extrinsic stress, including DNA damage, proteotoxicity, oxidative stress, nutrient deprivation, and exposure to chemotherapy (Chen et al. 2012, Gadgil et al. 2025, Kaya et al. 2015, Liu et al. 2015, Ly et al. 2011, Pavelka et al. 2010, Ravichandran et al. 2018, Rutledge et al. 2016, Sunshine et al. 2015, Yona et al. 2012). These findings suggest that under compromised or suboptimal growth conditions, aneuploidy may function as a mechanism of stress tolerance or adaptation. In the context of cancer, this implies that the selective advantages conferred by aneuploidy must outweigh its inherent proliferative cost, tipping the balance in favor of tumor evolution and survival (Figure 1).

This concept is further supported by studies demonstrating that the presence of extra chromosomes or elevated CIN can suppress characteristics associated with malignant transformation (Sheltzer et al. 2017). Engineered trisomic cells exhibit reduced fitness both in vitro and in vivo, although prolonged culture led to secondary chromosomal alterations that eventually improved their growth (Sheltzer et al. 2017). Similarly, mice with CIN-induced aneuploidy were predisposed to form spontaneous tumors in some tissues, yet this also suppressed tumor formation in others (Weaver et al. 2007). Together, these observations suggest that while chromosomal alterations may initially hinder tumorigenesis, their genome-destabilizing effects can eventually promote tumor evolution under selective pressure. Indeed, distinct patterns of chromosome gains and losses often emerge in tumors from CIN mouse models as subclones harboring specific aneuploidies become selectively enriched throughout tumorigenesis (Shoshani et al. 2021, Trakala et al. 2021).

**Figure 2:**
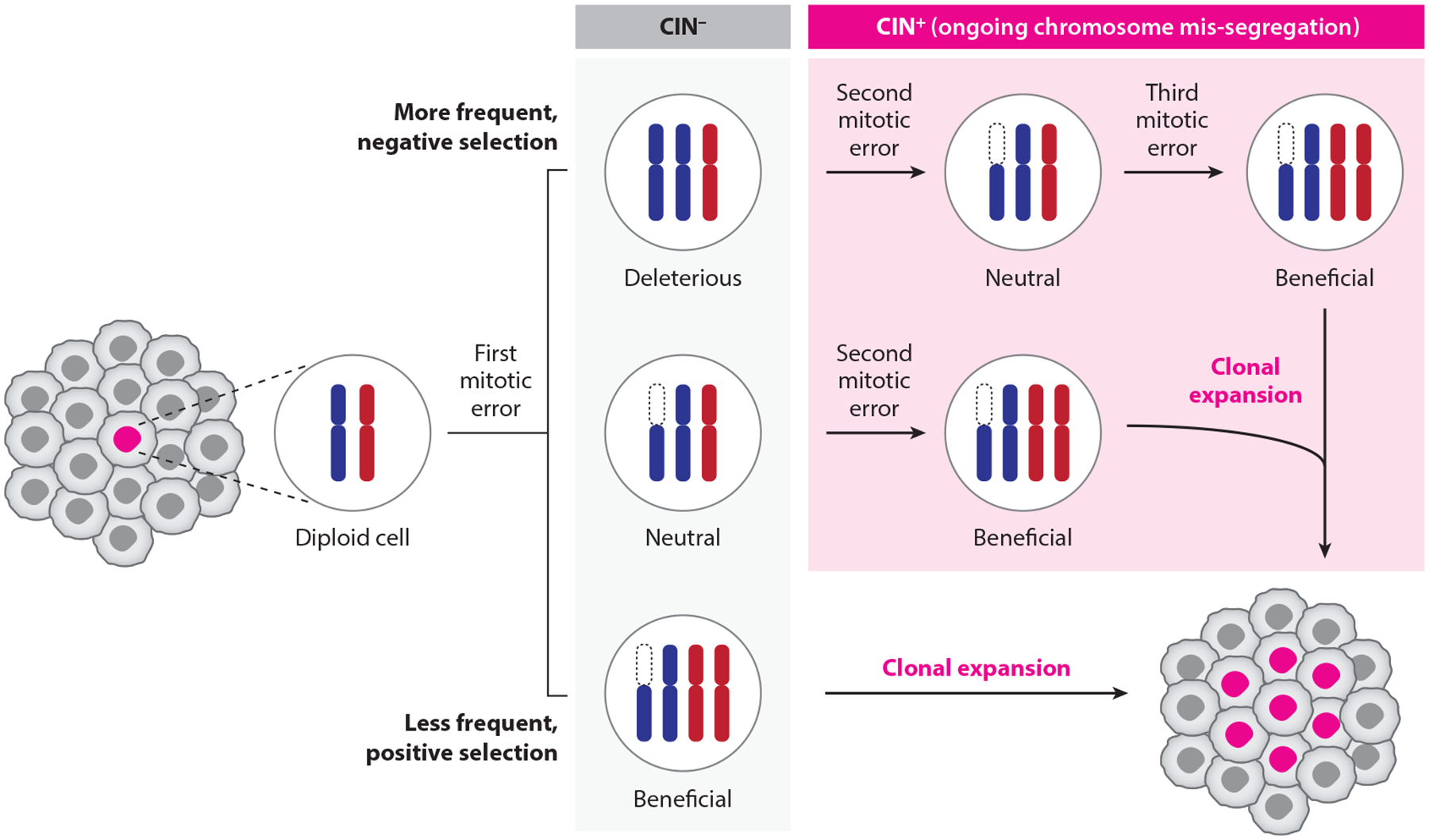
Chromosomal instability (CIN) drives karyotypic diversity throughout cancer genome evolution. Mitotic chromosome segregation errors can produce aneuploid daughter cells. Most aneuploidies are deleterious and subjected to negative selection. In chromosomally stable cells (CIN^−^), such deleterious karyotypes represent evolutionary dead ends as they do not contribute to further clonal expansion. However, chromosomally unstable cells (CIN+) exhibiting persistent mitotic errors can generate ongoing karyotypic diversity, thereby increasing the probability of the emergence of advantageous karyotypes that undergo positive selection and clonal outgrowth.

CIN allows cancer cells to continuously reshuffle their genomic content, much like a poker player granted unlimited opportunities to exchange cards in pursuit of a winning hand (an analogy initially described by D.W. Cleveland, unpublished). In a standard game of poker, players are allowed only one opportunity to exchange cards, with the outcome largely subject to chance. By contrast, CIN drives ongoing chromosomal gains and losses, generating a dynamic and heterogeneous pool of karyotypes (Figure 2). Although most changes are deleterious, some configurations may confer selective advantages—akin to eventually drawing a winning hand. This persistent genomic reshuffling increases the probability of acquiring karyotypes that support, rather than suppress, cancer development.

Cancer cells can also become strongly dependent on a specific aneuploidy for their maintenance or survival. For example, the targeted removal of a recurrent chromosome 1q gain in melanoma, gastric, and ovarian cancer cell lines abrogated their tumorigenic capacity (Girish et al. 2023). However, cells that successfully formed xenograft tumors regained an additional copy of chromosome 1q without evidence of acquiring other genomic alterations, underscoring the strong selective pressure to maintain this particular aneuploidy. Notably, the deletion of other nonrecurrent chromosomal gains did not impact tumorigenic potential to the same extent as chromosome 1q, suggesting that the oncogenic potential of aneuploidy depends on the identity of the chromosome and the context of the tumor (Girish et al. 2023). These findings imply that while aneuploidy imposes a general fitness cost, it can also provide strong selective advantages under specific genetic and environmental conditions.

### Adaptation via Whole-Genome Duplication

Following the bypass of cell cycle checkpoints in response to mitotic errors, how do cells eventually adapt to an aneuploid karyotype and/or tolerate CIN? A common strategy employed by cancer cells is whole-genome duplication (WGD), which generates a tetraploid (4N) genome. This most likely represents an intermediate state that buffers against chromosome mis-segregation by minimizing the relative impact of gaining or losing individual chromosomes. Indeed, monosomies are particularly detrimental to cellular fitness (Chunduri et al. 2021, Fusari et al. 2025), although they are better tolerated after WGD. In support of this, a pan-cancer analysis of human cancers found evidence of WGD in approximately one-third of cases, with whole-genome doubled tumors exhibiting abundant DNA copy number alterations with an average ploidy less than a full tetraploid state (~3.3N), suggesting extensive post-WGD chromosome loss (Zack et al. 2013).

WGD can arise through endoreduplication, mitotic slippage, or failed cytokinesis.While polyploidy is a normal feature of specific differentiated cell types (e.g., hepatocytes, megakaryocytes, trophoblasts), it is generally associated with reduced proliferative capacity (Cao et al. 2017, Gupta 2000, Mehrotra et al. 2008, Rios et al. 2016). In cancer, however, WGD may be a permissive event that facilitates genome plasticity and adaptation and is closely associated with increased aneuploidy and CIN (Bielski et al. 2018, Dewhurst et al. 2014, Fujiwara et al. 2005, Ganem et al. 2009, Gerstung et al. 2020, Minussi et al. 2021, Quinton et al. 2021, Watkins et al. 2020, Zack et al. 2013). WGD is also strongly correlated with *TP53* loss and *CCNE1* amplification, pointing to the role of these pathways in bypassing genome-doubling checkpoints and promoting oncogenic proliferation. Although it has been established that WGD promotes genomic instability, its precise impact on the patterns of aneuploidy observed across tumor types remains an open question.

## TARGETING ANEUPLOIDY AND CHROMOSOMAL INSTABILITY

### Exploiting Chromosomal Instability–Specific Vulnerabilities

The high prevalence of CIN and aneuploidy in cancer has prompted efforts to develop therapies that either exacerbate genome instability beyond a tolerable threshold or exploit vulnerabilities specific to the aneuploid state. Although SAC defects are known to promote CIN, mutations in core SAC genes are rare in human cancers (Jallepalli et al. 2001; Kops et al. 2004; Michel et al. 2001, 2004). Instead, SAC components are often overexpressed, indicating that many cancer cells preserve checkpoint function despite ongoing mitotic errors (Matthews et al. 2022, Pérez de Castro et al. 2007, Ryan et al. 2012). This preserved SAC activity appears essential, as inactivation of the checkpoint in cancer cells exhibiting CIN can result in catastrophic mitotic errors and cell death, suggesting that while low levels of CIN may be tolerated or even advantageous, excessive instability is lethal (Birkbak et al. 2011, de Cárcer et al. 2018, Janssen et al. 2009, Kops et al. 2004). Notably, even partial attenuation of SAC activity can sensitize aneuploid cells to chemotherapeutic agents (Janssen et al. 2009), revealing a potential therapeutic window.

Cancers with CIN often dysregulate mitotic kinases, such as Aurora A, Aurora B, and PLK1, which correlates with poor prognosis and further implicates mitotic fidelity as a critical vulnerability (Lens et al. 2010, Malumbres 2011, Sen et al. 2002, Sotillo et al. 2007). Inhibition of these kinases can exacerbate mitotic defects in CIN-positive cells, inducing cell death through mechanisms analogous to synthetic lethality. Accordingly, CIN-positive tumors display a heightened reliance on SAC integrity, rendering them particularly susceptible to therapies targeting mitotic fidelity (Cohen-Sharir et al. 2021, Quinton et al. 2021).

Rapidly proliferating, mitotically active cancer cells are clinically targeted by two classes of antimitotic drugs, vinca alkaloids (e.g., vinblastine, vinorelbine, vincristine) and taxanes (e.g., paclitaxel, docetaxel), both of which induce mitotic arrest by interfering with spindle microtubule dynamics. Live-cell imaging studies show that taxane-induced mitotic arrest is followed by either apoptosis or mitotic slippage into G1, the latter resulting in severe micronucleation and delayed cell death in interphase (Orth et al. 2011). Despite widespread success in cancer treatment, these agents remain limited by both intrinsic and acquired resistance, as well as dose-limiting toxicities. As a result, recent efforts have shifted toward developing more selective, mitosis-targeted therapies, including inhibitors of MPS1, PLK1, APC/C, and Aurora kinases (Mitchison 2012), although some have exhibited inadequate efficacy or toxicity to proliferative normal tissues in clinical trials (Komlodi-Pasztor et al. 2011).

Emerging studies have further nominated molecular motor proteins as promising targets. This is exemplified by KIF18A, a plus-end-directed kinesin required for proper metaphase chromosome alignment. Large-scale genetic screens identified *KIF18A* as a selectively essential gene in cells with high CIN, suggesting a potential dependency due to a heightened reliance on accurate chromosome segregation (Cohen-Sharir et al. 2021, Quinton et al. 2021). Indeed, inhibition of KIF18A robustly impairs the viability of cancer cells exhibiting CIN while sparing nontransformed, chromosomally stable cells (Cohen-Sharir et al. 2021, Gliech et al. 2024, Marquis et al. 2021, Phillips et al. 2025, Quinton et al. 2021). Early-phase KIF18A inhibitors are currently under evaluation in solid tumors, with additional molecules in preclinical development (Mohd Amin et al. 2024).

### Targeting Recurrent Aneuploidies

Beyond strategies targeting CIN or mitotic processes, there is growing interest in exploiting the selective vulnerabilities conferred by specific aneuploidies (Bhatia et al. 2024, Shukla et al. 2020). A well-characterized example is the loss of one copy of chromosome 3p in ccRCC, which results in the deletion of multiple tumor suppressor genes, including the E3 ubiquitin ligase *VHL* (encoding for pVHL) (Gerlinger et al. 2012, 2014; Mitchell et al. 2018; Zbar et al. 1987). Somatic mutation of the remaining *VHL* allele leads to its biallelic inactivation and subsequent stabilization of the ccRCC-specific oncogenic driver HIF-2α. This creates a dependency that has been successfully targeted in the clinic with selective HIF-2α inhibitors (e.g., belzutifan) (Chen et al. 2016, Cho et al. 2016).

Such chromosome-scale deletions can also create opportunities for collateral lethality, a concept in which the codeletion or dosage reduction of nearby essential genes renders tumor cells reliant on compensatory mechanisms (Muller et al. 2015). For example, in tumors with chromosome 9p21 loss encompassing the *CDK2NA* locus, the adjacent *MTAP* gene is frequently codeleted, leading to the accumulation of a metabolite that selectively inhibits PRMT5 methyltransferase activity and creates a partial loss-of-function state. This metabolic vulnerability renders MTAP-deleted cells dependent on PRMT5 and its cofactors, identifying a targetable axis for therapeutic intervention in 9p21-deleted tumors (Marjon et al. 2016, Mavrakis et al. 2016). In ccRCCs with chromosome 3p loss in which hundreds of genes are codeleted, there may exist additional yet-uncharacterized vulnerabilities beyond the canonical pVHL–HIF-2α signaling pathway.

Similarly, cancers with loss of chromosome 2q, which encompasses the essential splicing factor *SF3B1*, are sensitized to spliceosome inhibitors, highlighting a nononcogene addiction that arises from aneuploidy-induced haploinsufficiency (Paolella et al. 2017). In breast cancer, loss of chromosome 8p correlates with upregulation of autophagy-related pathways and increased sensitivity to autophagy inhibitors (Cai et al. 2016, Tschaharganeh et al. 2016). Conversely, the recurrent gain of chromosome 1q results in the coamplification of *UCK2*, a pyrimidine salvage kinase involved in nucleoside processing. The collateral amplification of UCK2 thus creates a dependency that renders cancer cells particularly sensitive to nucleoside analog-based therapies (Girish et al. 2023), illustrating a synthetic dosage lethality mechanism whereby overexpression of one gene creates a vulnerability upon targeting of a cooperating amplified gene. A final example stems from the recurrent amplification of chromosome 17q23 in breast cancer and neuroblastoma (Andersen et al. 2002), which drives the overexpression of numerous genes, including the E3 ubiquitin ligase *TRIM37*. Elevated TRIM37 levels impair centrosome-independent spindle assembly by degrading components of the pericentriolar material, in turn rendering sensitivity to PLK4 inhibition, which prevents centrosome duplication (Meitinger et al. 2020, Yeow et al. 2020). This context-specific dependency exemplifies how aneuploidy-induced gene dosage imbalances can uncover collateral vulnerabilities that are selectively targetable in cancer.

### Genetic Dependencies and Synthetic Lethality

Synthetic lethality—where the combined perturbation of two genes leads to cell death but alteration of either alone is tolerated—provides a conceptual basis for identifying cancer-selective vulnerabilities (Hartwell et al. 1997). This principle underlies the clinical success of PARP inhibitors in homologous recombination-deficient tumors (Bryant et al. 2005). Similar approaches are now being explored in the context of CIN. For instance, hepatocellular carcinomas with chromosome 8p loss are dependent on *NUDT17*, a paralog of *NUDT18*, which is encoded on 8p and normally contributes to detoxification of oxidized nucleotides. Loss of *NUDT18* creates a synthetic lethal dependency on *NUDT17*, suggesting a therapeutic opportunity (Huth et al. 2023). In ovarian cancers with amplification of chromosome 19q12 resulting in overexpression of cyclin E1, a driver of replication stress, cells are sensitized to inhibition of PLK1 and PKMYT1, kinases that are involved in cell cycle progression and checkpoint regulation (Gallo et al. 2022, Noack et al. 2018, Xi et al. 2025). While promising, significant challenges remain in translating these discoveries into effective therapies, including identifying actionable genetic dependencies in heterogeneous tumor populations, developing selective inhibitors, and overcoming drug resistance (Lin & Sheltzer 2020).

## CONCLUDING REMARKS

In conclusion, aneuploidy and CIN are defining features of cancer genomes, yet their biological consequences remain paradoxical. While both processes can impair cellular fitness and viability, they also generate the genomic diversity that is required to enable tumor evolution and progression. Recent advances have begun to uncover how cancer cells adapt to the burdens imposed by aneuploidy through diverse mechanisms, such as WGD, activation of stress response pathways, and selective pressure for compensatory mutations. These adaptations allow for continued proliferation while also exposing potential vulnerabilities that can be therapeutically exploited. Indeed, there is growing interest in translating these mechanistic insights into clinical strategies, including targeting mitotic regulators, proteotoxic stress pathways, and aneuploidy-specific dependencies. Despite ongoing challenges associated with tumor heterogeneity, toxicity, and resistance, accumulating evidence indicates that aneuploidy and CIN are not merely byproducts of cancer but instead actively contribute to its development. Future studies will continue to explore how cells tolerate and evolve under conditions with an unbalanced genome, which will uncover new foundational principles underlying cancer biology and potential therapeutic opportunities.
